# Preoperative Cardiac Risk Stratification in Dogs with Mammary Tumors Using Two-Dimensional Speckle Tracking Echocardiography: A Pilot Study

**DOI:** 10.3390/ani16091409

**Published:** 2026-05-04

**Authors:** Didem Algan, Tuğba Varlik, Hüseyin Tan, Pelin Erden, Lina Hamabe, Ryou Tanaka, Zeki Yilmaz

**Affiliations:** 1 Department of Internal Medicine, Veterinary Faculty, Bursa Uludag University, Bursa 16059, Türkiye; didem.algann@gmail.com (D.A.); tugbav.vet@gmail.com (T.V.); zyilmaz@uludag.edu.tr (Z.Y.); 2 Department of Statistics, Middle East Technical University, Ankara 06800, Türkiye; hhuseyintan@outlook.com.tr; 3 Department of Obstetrics and Gynecology, Veterinary Faculty, Bursa Uludag University, Bursa 16059, Türkiye; pelinerden@uludag.edu.tr; 4 Department of Veterinary Medicine, Faculty of Agriculture, Tokyo University of Agriculture and Technology, Fuchu 183-8538, Japan; fu0253@go.tuat.ac.jp

**Keywords:** canine mammary tumors, speckle-tracking echocardiography, machine learning, risk assessment

## Abstract

Although breast cancer has been associated with subclinical left ventricular dysfunction in humans, the cardiac effects of CMTs remain poorly defined. This pilot, exploratory (communication) study compared clinical and echocardiographic parameters between dogs with CMTs and healthy controls and assessed the feasibility of combining myocardial deformation imaging with exploratory data-driven analysis for preoperative cardiac assessment. All dogs underwent a standardized clinical and echocardiographic assessment, including two-dimensional speckle-tracking echocardiography (2D-STE). Given the limited sample size, analyses were designed to generate hypotheses rather than to provide definitive predictive conclusions. Exploratory machine learning modeling (XGBoost), receiver operating characteristic (ROC) analysis, calibration, and decision curve analysis were performed as proof-of-concept approaches without external validation. Despite normal conventional systolic indices, dogs with CMTs exhibited reduced global longitudinal strain (GLS) and mitral annular plane systolic excursion (MAPSE) (*p* < 0.01), suggesting subclinical systolic dysfunction. Deformation-derived parameters appeared more sensitive for detecting subtle myocardial alterations within this cohort. The exploratory machine learning model demonstrated moderate discrimination (AUC-ROC = 0.75); however, these findings are preliminary and should not be interpreted as evidence of clinical predictive performance. Overall, these results suggest that conventional systolic indices may underestimate early myocardial changes in dogs with CMTs. This communication highlights the feasibility of integrating deformation imaging with exploratory analytical approaches and provides a basis for future large-scale, validated studies in veterinary cardio-oncology.

## Simple Summary

Mammary tumors are among the most common cancers in female dogs and typically affect middle-aged to older animals. This population is also at an increased risk of heart disease. In human medicine, breast cancer has been linked to subtle heart dysfunction even before clinical signs appear. However, the potential impacts of naturally occurring canine mammary tumors (CMTs) on the structure and function of the heart in dogs remain unclear. In this pilot hypothesis-generating study, we compared heart function in dogs with CMTs and in healthy control dogs using advanced echocardiographic techniques. Although conventional measurements were normal, dogs with CMTs showed reduced longitudinal heart function, suggesting possible early subclinical myocardial impairment. Parameters such as mitral annular plane systolic excursion (MAPSE) and global longitudinal strain (GLS) appeared sensitive in detecting these differences. An exploratory machine learning approach was also applied to assess methodological feasibility rather than to establish a predictive model. Due to the small sample size, the findings should be interpreted as preliminary and require confirmation in larger studies. These results provide initial evidence that myocardial deformation imaging may detect subtle cardiac alterations in dogs with CMTs and support further investigation within a comparative cardio-oncology and One Health perspective.

## 1. Introduction

Mammary tumors are the most prevalent neoplasms in intact female dogs worldwide and the second most common tumor type after cutaneous neoplasms. Incidence increases with age, particularly beyond six years, peaking between 8 and 11 years [1]. Breed predisposition (especially among miniature and toy breeds) along with obesity and dietary factors such as high red meat consumption further contribute to risk, underscoring both genetic and environmental influences in mammary tumorigenesis [2,3,4]. Histopathology remains the diagnostic gold standard, and surgery is the primary treatment modality, except in cases of inflammatory carcinoma, where medical management is generally preferred. Although surgical approaches range from local excision to radical mastectomy, the optimal extent of resection remains a subject of debate [4,5,6].

Affected dogs typically belong to an age group inherently predisposed to cardiovascular dysfunction. In human oncology, tumor-associated systemic inflammation, oxidative stress, neurohormonal activation, and prothrombotic pathways contribute to subclinical myocardial remodeling independent of overt cardiotoxic therapy, forming the basis of cardio-oncology [7,8,9]. These systemic cancer-related effects may impair myocardial mechanics before clinical cardiac disease becomes apparent. However, the cardiovascular implications of naturally occurring tumors in dogs remain insufficiently characterized.

Cardiac complications represent a significant source of perioperative morbidity and mortality in non-cardiac surgery, emphasizing the need for preoperative cardiac risk stratification [7,10]. Current veterinary guidelines recommend electrocardiographic (ECG) evaluation in geriatric patients with suspected cardiac disease; nevertheless, the clinical impact of routine pre-anesthetic cardiac screening on anesthetic management remains uncertain [11,12]. Conventional echocardiographic indices such as fractional shortening (FS%) and ejection fraction (EF%) reflect global systolic performance and may fail to detect subtle longitudinal dysfunction [13]. Therefore, more sensitive imaging-based markers are needed to identify early myocardial impairment before overt systolic dysfunction develops.

Two-dimensional speckle-tracking echocardiography (2D-STE) provides angle-independent quantification of myocardial deformation [8,9] and has demonstrated superior sensitivity in detecting early functional alterations compared with conventional echocardiographic parameters in dogs [13,14]. Global longitudinal strain (GLS) and mitral annular plane systolic excursion (MAPSE) are particularly sensitive markers of subclinical systolic impairment [15,16].

In parallel, recent advances in machine learning (ML) have expanded the potential for cardiovascular risk prediction through the integration of high-dimensional clinical and imaging data. Algorithms such as eXtreme Gradient Boosting (XGBoost) can capture complex non-linear relationships, optimize feature selection and improve individualized risk stratification. The integration of SHapley Additive exPlanations (SHAP) further improves model interpretability by quantifying the relative contribution of each predictor to the overall risk estimate, thereby supporting precision-based cardiovascular assessment [7,15,17].

Accordingly, the present study was designed as a pilot, hypothesis-generating investigation to explore subclinical myocardial changes in dogs with mammary tumors using 2D-STE. In addition, an exploratory ML-based framework was applied to assess the feasibility of integrating deformation-derived parameters with conventional clinical variables, rather than to establish a validated predictive model. We hypothesized that dogs with mammary tumors may exhibit subtle impairment in longitudinal systolic function despite preserved conventional indices. Furthermore, we aimed to explore whether combining deformation imaging with interpretable ML approaches could provide preliminary insights into perioperative cardiac assessment. Given the limited sample size, the findings are intended to be exploratory and to inform future, larger-scale studies in veterinary cardio-oncology.

## 2. Materials and Methods

The study was approved by the Institutional Animal Care and Use Committee (Bursa Uludag University Local Ethics Committee; 2026-03/05 24.02.2026).

### 2.1. Study Population

This study included a total of 16 client-owned dogs, retrospectively enrolled from the medical records of the Department of Internal Medicine (Faculty of Veterinary Medicine, Bursa Uludag University, Bursa—Türkiye). All dogs underwent a standard physical examination and cardiologic evaluation (ECG and echocardiography), as well as laboratory analysis (complete blood cell count and serum biochemistry). All diagnostic procedures were part of routine clinical evaluation.

The dogs were divided into two groups: cardiologically healthy dogs served as the control group (n = 7), and dogs diagnosed with mammary tumors by either the Department of Obstetrics and Gynecology or private animal hospitals constituted the study group (n = 9). Clinically healthy dogs with no clinical signs of cardiac disease and no echocardiographic evidence of structural or functional cardiac abnormalities were classified as the control group.

### 2.2. Transthoracic Echocardiography

For preoperative risk assessment, all dogs underwent transthoracic echocardiographic examination (Vivid S60N Ultra Edition, GE Healthcare, Horten, Norway; Penta Electronic, distributor in Istanbul, Türkiye) without sedation using a multi-frequency phased-array transducer appropriate for canine cardiac imaging (6S or 3S cardiac probes). Echocardiographic measurements were obtained during quiet respiration and averaged from three consecutive cardiac cycles. Dogs were examined in right and left lateral recumbency. Cardiac imaging included conventional two-dimensional (2D), M-mode, and Doppler echocardiography using standard imaging planes, including right parasternal short-axis (RPSAx) and long-axis (RPLAx) views, as well as left apical two-, three-, and four-chamber views, in accordance with established canine echocardiographic guidelines [18]. All images and video recordings were stored and subsequently transferred to a computer equipped with analysis software (EchoPac™ PC, GE HealthCare, version 204, Horten, Norway), to conduct all measurements offline, as reported in our previous studies [19,20].

#### 2.2.1. Conventional Echocardiography

Standard echocardiographic measurements, including left ventricular (LV) internal dimensions at end-diastole (LVIDd) and end-systole (LVIDs), as well as interventricular septal thickness (IVSd) and left ventricular posterior wall thickness at end-diastole (LVPWd) and end-systole (IVSs and LVPWs, respectively), were measured from the RPSAx view at the level of the papillary muscle using M-mode echocardiography. The normalized LV internal diameter in diastole (LVIDDN) was calculated using the formula: LVIDDN = LVIDd (cm)/(body weight (kg)^0.294^) [21]. Fractional shortening (FS) and ejection fraction (EF) were calculated automatically (Teichholz method) following the M-mode measurements of the LV at RPSAx at the papillary muscle level. Also, LV volume was calculated using the auto ejection fraction (AutoEF) function.

Left atrial (LA) and aortic (Ao) diameters were measured at end-systole from the RPSAx view at the level of the heart base. The LA diameter was defined as the distance from a line drawn parallel to the commissure between the non-coronary and left coronary Ao valve cusps to the dorsal wall of the LA. The Ao diameter was measured along the commissure between the non-coronary and left coronary cusps, bisecting the right coronary cusp, and the LA/Ao ratio was subsequently calculated. Pulsed-wave (PW) Doppler echocardiography was additionally performed from the left apical four-chamber view to assess the peak velocity of early diastolic LV filling (E-wave) and late atrial contraction (A-wave) [22].

#### 2.2.2. Two-Dimensional Speckle Tracking Echocardiography (2D-STE)

Following conventional echocardiographic assessment, myocardial deformation analysis was performed using 2D-STE. Grayscale image settings were carefully optimized to clearly delineate endocardial borders and maximize tracking accuracy. Frame rates ranged from approximately 90 to 124 frames per second, depending on heart rate and image optimization. For each dog, three to five consecutive cardiac cycles were recorded from the apical four-chamber (A4C) view, with simultaneous one-channel ECG monitoring to allow precise temporal alignment of cardiac events. R–R interval-gated strain analysis was performed for six LV segments (septal and lateral base, mid, and apex). For each dog, GLS values were derived from a single optimized cardiac cycle [8,23]. To ensure reliability, multiple cine-loops were reviewed for image quality, tracking stability, and ECG consistency. Although three consecutive cardiac cycles were acquired, the final GLS value was selected from the cardiac cycle that demonstrated optimal tracking quality, minimal motion artefacts, and stable strain curves throughout systole and diastole [8].

LV longitudinal systolic function was additionally evaluated using the tissue tracking (TT) module. TT represents the time integral of myocardial tissue velocity as the distance of motion along the ultrasound beam axis, thereby providing a quantitative measure of longitudinal myocardial displacement. From an LV-focused apical four-chamber view, tissue velocity imaging (TVI) cine loops were acquired at a frame rate of >90 fps and stored for offline analysis. Event timing was ECG-guided and based on PW Doppler-derived aortic and mitral flow profiles to define valve opening and closure. Within the predefined systolic interval, longitudinal displacement values were calculated at the septal (TTsep) and lateral (TTlat) segments of the mitral annulus [20]. In addition, short-axis views at the level of the papillary muscles were used to calculate global circumferential strain (GCS) and circumferential strain rate (GCSr).

### 2.3. Inclusion and Exclusion Criteria

Healthy control dogs were retrospectively identified from hospital records between 2023 and 2025. These included dogs presented for comprehensive health evaluations at the owners’ request. Dogs showing no clinical, hematological, or echocardiographic abnormalities were included in the control group, as previously described [8,22].

Dogs were classified as healthy controls if they fulfilled all of the following echocardiographic inclusion criteria [22]:

- Normal LV wall thickness (IVSd and LVPWd at end-diastole) was defined within body weight-adjusted reference ranges using M-mode echocardiography from the RPSAx view at the papillary muscle level, based on canine allometric scaling.
- Normal LV chamber dimensions, including LVIDd and LVIDs, with values falling within predicted normal limits after normalization for body weight (LVIDDN).
- Preserved global systolic function, defined as FS% within the normal reference range for dogs (>25%), with no echocardiographic evidence of systolic dysfunction.
- Normal LA size, defined as a LA/Ao ratio ≤ 1.3, measured from the RPSAx view at the level of the aortic valve, with no evidence of LA enlargement.
- Physiological diastolic filling pattern, defined by normal early diastolic transmitral inflow velocity (E-wave) assessed by PW Doppler echocardiography from the left apical four-chamber view, without evidence of diastolic dysfunction.
- Absence of structural cardiac disease, including congenital cardiac anomalies, valvular dysplasia, myocardial hypertrophy or dilation, regional wall motion abnormalities, or pericardial effusion on two-dimensional and Doppler echocardiographic examination.
- Normal cardiac rhythm, defined as sinus rhythm documented during echocardiographic acquisition, with exclusion of dogs presenting atrial or ventricular arrhythmias.
- Normal myocardial deformation parameters, defined as GLS values derived from the apical four-chamber view using 2D-STE within previously reported physiological ranges for dogs, with homogeneous segmental strain distribution and no evidence of subclinical myocardial dysfunction.

Cases with mammary tumors were excluded from the study if they had any concurrent systemic infection, endocrine disorder (including diabetes mellitus or hyperadrenocorticism), renal insufficiency, or evidence of hepatic or pancreatic injury. Upon review of the medical records of the selected cases, patients lacking adequate echocardiographic image quality—thereby potentially yielding unreliable or questionable results—were also excluded from the study.

### 2.4. Statistical Analysis

Statistical analyses were performed to compare healthy dogs and dogs with mammary tumors based on clinical and echocardiographic parameters. Data distribution was assessed using the Shapiro–Wilk test. Normally distributed variables are expressed as the mean ± standard deviation (SD), whereas non-normally distributed variables are presented as the median (interquartile range, IQR). Between-group comparisons were conducted using the independent samples t-test for parametric data and the Mann–Whitney U test for non-parametric data. A two-tailed *p*-value < 0.05 was considered statistically significant.

To evaluate the distributional characteristics and group separation of significant variables, Kernel Density Estimation (KDE) plots were generated. Violin plots combined with box plots were used to provide a comprehensive visualization of central tendency, dispersion (IQR), and potential outliers for the parameters identified as significant. Linear relationships among echocardiographic variables were assessed using correlation analysis, and results were visualized through correlation heatmaps. Correlation coefficients (*r*) were calculated, and only statistically significant associations (*p* < 0.05) were displayed in the filtered heatmap to eliminate stochastic relationships and highlight biologically meaningful dependencies. In addition, the relationship between frame rate and GLS was specifically evaluated using Spearman correlation analysis.

To explore potential associations between longitudinal functional parameters and clinical variables, multivariable linear regression analyses were performed. Separate models were constructed using MAPSE and GLS as dependent variables. Age, body weight, heart rate, and LVIDDN were included as independent variables based on prior evidence. Model assumptions, including normality, homoscedasticity, and multicollinearity, were evaluated. Given the limited sample size, these analyses were considered exploratory, and the results were interpreted cautiously without inferring independent predictive relationships.

To further explore pattern within the dataset, a supervised ML approach was implemented using eXtreme Gradient Boosting (XGBoost). The ML analysis was conducted as an exploratory, proof-of-concept approach to assess feasibility rather than to develop or validate a predictive model [24]. The model was trained to classify dogs as having tumors (1) or being healthy (0), and performance metrics including precision, recall (sensitivity), area under the receiver operating characteristic curve (AUC-ROC), and the Gini coefficient (Gini = 2 × AUC − 1) were calculated. Given the small sample size and absence of external validation, these performance metrics are reported descriptively and should not be interpreted as evidence of clinical predictive utility or generalizability. No internal data partitioning (e.g., training–test split) or resampling techniques (such as k-fold cross-validation or bootstrapping) were employed. As a result, model performance was assessed on the same dataset used for model development, introducing a risk of optimistic bias and limiting the robustness and generalizability of the reported metrics.

To enhance interpretability, SHAP analysis was performed. SHAP values were used to quantify the direction and magnitude of each variable’s contribution to the prediction. Positive SHAP values indicated an increased probability of belonging to the tumor group, whereas negative values supported classification in the healthy group. SHAP results were used to provide preliminary insights into feature importance within this dataset and should be interpreted as hypothesis-generating. Robust validation strategies, including k-fold cross-validation or external validation, were not feasible due to the limited sample size, which represents a key limitation of the analytical framework.

All statistical and machine learning analyses were performed using appropriate statistical software and Python-based machine learning (version of Python 3.12.11) libraries as reported in our previous study [17].

## 3. Results

Both groups included female dogs of various breeds and ages (5–11 years). Dogs with mammary tumors were significantly older than healthy controls (Table 1). None of the animals exhibited overt clinical signs of cardiac disease at the time of examination. The tumor group included terriers (n = 3), one Golden Retriever, one Pug, one Pointer, and mixed-breed dogs (n = 3). The control group was breed-matched and comprised the same number of dogs within each breed category.

### 3.1. Conventional Echocardiographic Measurements

**Figure 1:**
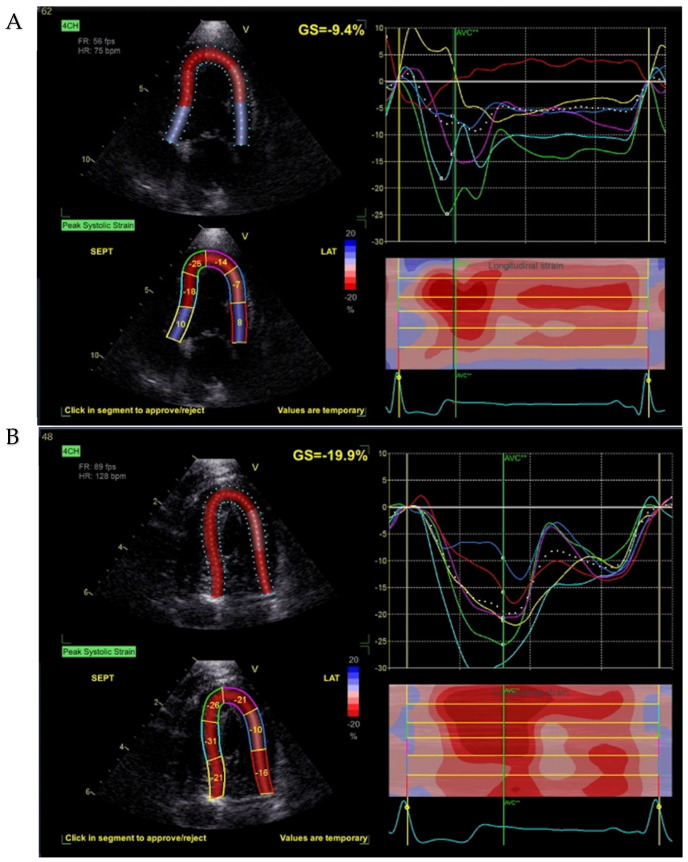
Global longitudinal strain (GLS) assessment using two-dimensional speckle-tracking echocardiography (2D-STE) from the apical four-chamber (A4C) view. Representative images from a dog with a mammary tumor (**A**) and a healthy control (**B**). Colored curves represent segmental longitudinal strain of the six left ventricular segments. GLS values are displayed for each example.

Conventional echocardiographic indices of global systolic function, including FS and EF, did not differ significantly between groups (*p* > 0.05) (Table 1). Similarly, LV dimensions, IVS thickness, LVPW thickness, and LA size (LA diameter, LA/Ao ratio, and LA volume) were comparable between groups (all; *p* > 0.05). Mitral and tricuspid inflow indices (E/A ratios), aortic and pulmonary peak velocities, tissue Doppler systolic velocities (TDI S′ septal and lateral), and heart rate did not differ significantly between groups (all; *p* > 0.05) (Table 1 and Figure 1).

### 3.2. Longitudinal Function and Deformation Analysis

**Figure 2:**
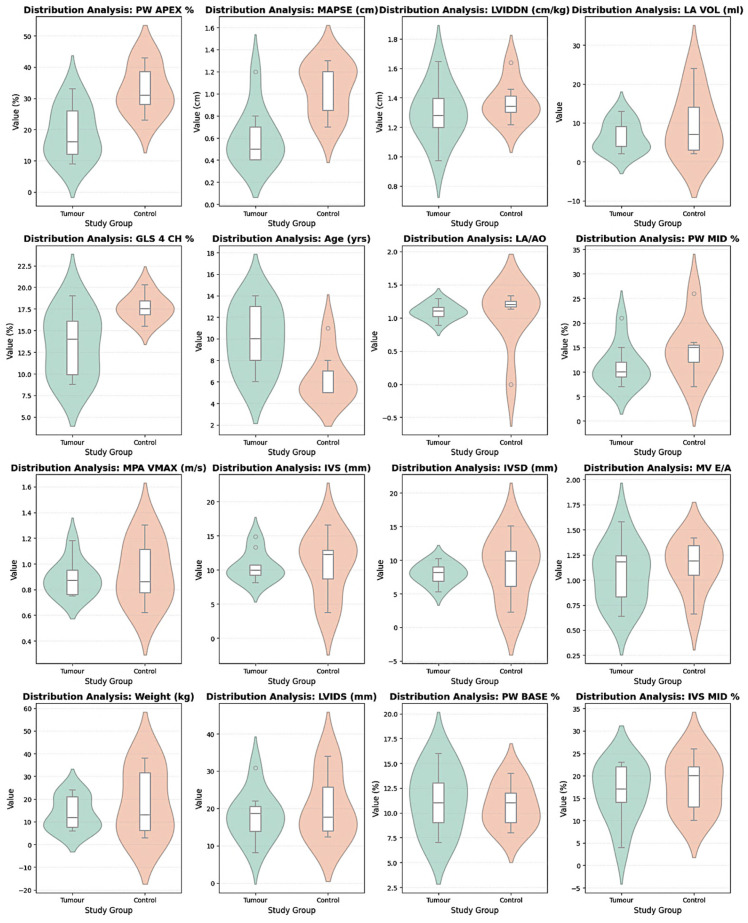
Violin plots of parameters with significant differences between groups. Box plots indicate median and interquartile range.

MAPSE was significantly reduced in the tumor group (0.6 ± 0.2 cm vs. 1.0 ± 0.2 cm, *p* < 0.01). GLS (4-chamber view) was also significantly lower in dogs with mammary tumors (−13.4 ± 3.7% vs. −17.6 ± 1.5%, *p* < 0.01) (Figure 1). Segmental strain analysis revealed a marked reduction in apical posterior wall strain (PW apex %) in the tumor group (−19.0 ± 8.3% vs. −32.8 ± 7.7%, *p* < 0.01) (Figure 2). Basal and mid-segmental strain values did not differ significantly between the groups. Analysis of global circumferential strain (GCS) and circumferential strain rate (GCSr) did not reveal significant differences between tumor-bearing and healthy dogs (Table 1). The mean ± SD frame rate was 92 ± 15 fps for the tumor group and 98 ± 12 fps for healthy controls. The potential influence of frame rate on GLS was examined, and no significant effect was observed.

Multivariable linear regression analyses showed that, after adjustment for age, body weight, heart rate, and LVIDDN, no variables were identified as independent predictors of either MAPSE or GLS; the MAPSE model demonstrated a high R^2^ (0.89), whereas the GLS model showed lower explanatory power (R^2^ = 0.30) and evidence of multicollinearity.

### 3.3. Correlation Analysis

**Figure 3:**
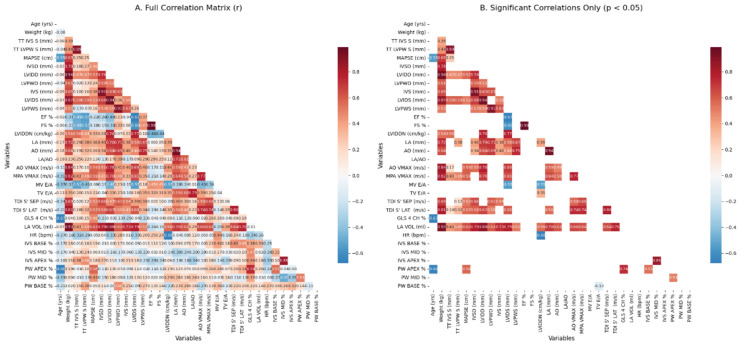
Correlation heatmaps of echocardiographic parameters. (**A**) Full correlation matrix. (**B**) Significant correlations (*p* < 0.05).

Correlation heatmap analysis revealed physiologically consistent relationships among longitudinal functional parameters (Figure 3). MAPSE showed a positive correlation with GLS and regional strain indices, whereas impaired strain parameters were inversely associated with age. After applying a significance threshold (*p* < 0.05), only robust biological associations were retained, confirming internal consistency within the dataset.

### 3.4. Machine Learning Performance and Discrimination

An XGBoost classification model achieved an AUC-ROC of 0.75 (Gini = 0.50), with a precision of 0.55 and recall of 0.85. Comparative ROC analysis demonstrated superior discrimination compared with individual conventional parameters.

**Figure 4:**
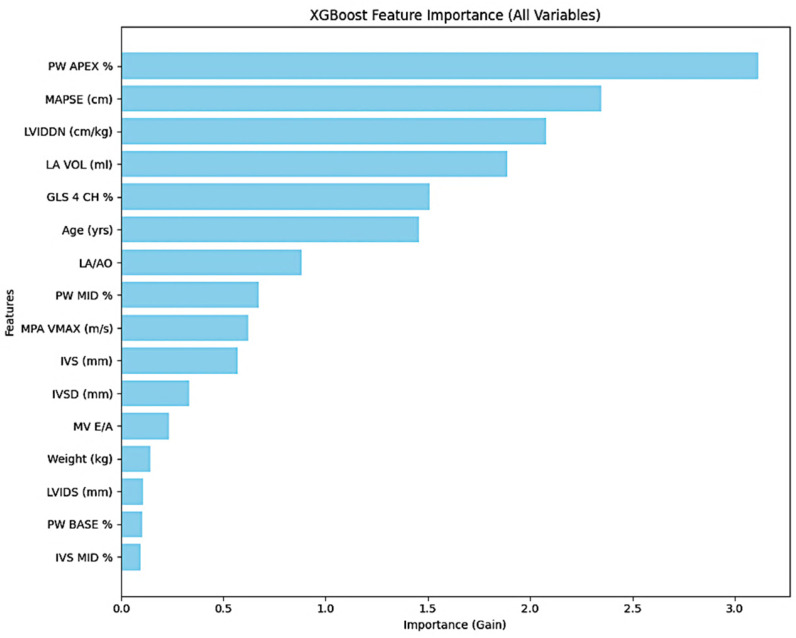
Feature importance plot from the XGBoost model showing the relative contribution of echocardiographic and clinical variables based on the gain metric.

Feature importance analysis based on gain identified PW APEX %, MAPSE (cm), LVIDDN (cm/kg), and LA VOL (mL) as the top contributing variables (Figure 4).

**Figure 5:**
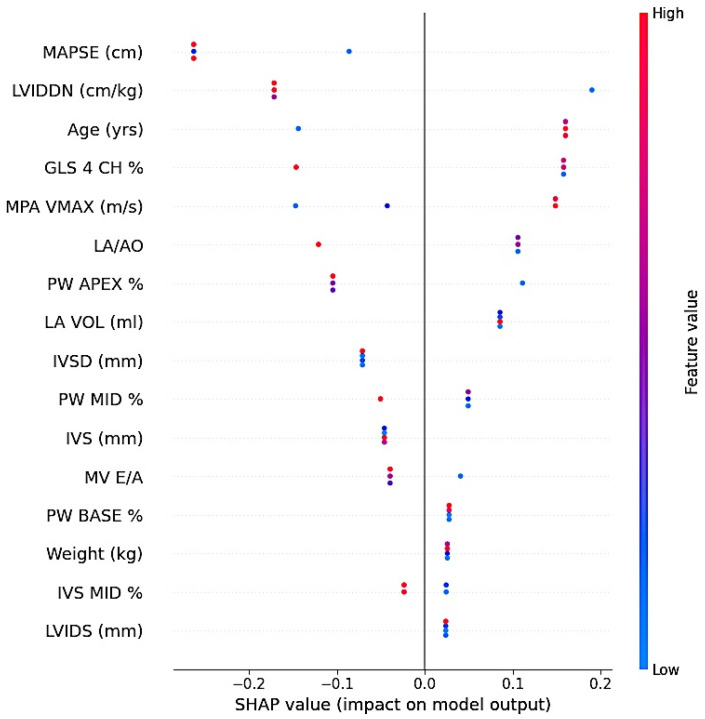
SHAP summary plot showing the contribution of variables to the XGBoost model. Each point represents a single observation. Color denotes feature value (red: high, blue: low), and SHAP values indicate the direction and magnitude of each variable’s contribution to the model output.

Ranking based on the Gain metric showed MAPSE as the highest contributor, followed by LVIDDN, age, and GLS. SHAP analysis indicated that lower MAPSE values, higher LVIDDN values, older age, and impaired GLS values were associated with classification into the tumor group. Variables with lower relative importance included body weight, mid-septal strain, and basal posterior wall strain (Figure 5).

### 3.5. Calibration and Clinical Utility

Calibration analysis showed agreement between predicted and observed probabilities. The calibration slope approached unity, and the Brier score indicated overall prediction accuracy. Decision Curve Analysis (DCA) showed a higher net benefit of the XGBoost model compared with “treat-all” and “treat-none” strategies, as well as individual conventional echocardiographic parameters across a range of threshold probabilities.

## 4. Discussion

The present study provides preliminary evidence suggesting that dogs with mammary tumors exhibit subclinical impairment of longitudinal LV function despite preserved conventional systolic indices. Although EF, FS, chamber dimensions, and Doppler-derived parameters did not differ significantly between groups, deformation imaging identified differences in GLS and MAPSE. Rather than indicating definitive myocardial remodeling, these findings should be interpreted as exploratory observations that may reflect early functional alterations. The integration of these variables into a machine learning framework was therefore considered a feasibility assessment rather than a validation of predictive performance.

In this study, circumferential strain (GCS) and circumferential strain rate (GCSr) remained comparable between tumor-bearing and healthy dogs, whereas reductions in longitudinal strain (GLS) and MAPSE were evident in the tumor group. This selective impairment of longitudinal mechanics supports the notion that early subclinical myocardial dysfunction predominantly involves subendocardial fibers, while mid-wall circumferential fibers are largely preserved, consistent with prior human and veterinary investigations [8,13,25,26]. Conventional echocardiographic indices, such as EF and FS, primarily reflect radial contraction and global volumetric changes, and may fail to detect subtle myocardial compromise until more advanced stages [13,25]. Accordingly, the observed differences in longitudinal parameters should be interpreted as potential indicators of early functional variation rather than confirmed dysfunction. While GLS and MAPSE differed between groups, their clinical and prognostic significance remains uncertain in the absence of outcome data.

Dogs in the tumor group were older and exhibited higher LVIDDN, reduced MAPSE, and less negative GLS values compared with controls. Age is a well-recognized determinant of myocardial deformation [27], and the significant age difference between groups represents an important limitation that cannot be fully controlled in the present study. Although multivariable regression models included age and other covariates, no independent predictors of MAPSE or GLS were identified. Given the small sample size and evidence of multicollinearity, these analyses are underpowered and should be interpreted cautiously. Therefore, it is not possible to distinguish whether the observed differences are attributable to tumor-related effects, age-related remodeling, or a combination of both. In human oncology, systemic inflammation, endothelial dysfunction, oxidative stress, and neurohormonal activation have been implicated in cancer-related myocardial remodeling independent of overt cardiotoxic therapy [7]. Whether similar mechanisms operate in dogs with naturally occurring mammary tumors remains speculative and was not directly investigated in this study.

Among the evaluated variables, MAPSE and GLS showed the largest between-group differences. MAPSE is a simple and widely accessible measure that correlates with longitudinal myocardial function [15,16,28]. Its reduction in tumor-bearing dogs, despite preserved conventional indices, supports its potential utility as a practical marker of early dysfunction. GLS also demonstrated strong discriminatory capacity, further reinforcing the importance of longitudinal functional assessment in this population. This finding is consistent with prior canine studies demonstrating that STE-derived strain parameters detect early LV dysfunction before conventional indices become abnormal [20,29]. However, the observed changes in MAPSE and GLS within the model should be interpreted as hypothesis-generating rather than indicative of stable or generalizable predictors. Although frame rate variability was considered in the analysis, the relatively broad range may still contribute to variability in GLS measurements and should be acknowledged as a methodological limitation. Segmental analysis identified lower apical posterior wall strain in tumor-bearing dogs; however, this regional pattern requires confirmation in larger cohorts before any physiological interpretation can be made.

Additional echocardiographic variables, including LA/Ao ratio, left atrial volume, septal thickness, and diastolic indices, showed modest contribution within the model. Although these parameters did not independently distinguish groups at a statistically robust level, their intermediate contribution suggests subtle structural and diastolic interactions accompanying longitudinal impairment. However, these observations should be interpreted cautiously and do not imply causal or independent relationships. Mild variations in atrial size and diastolic filling patterns were observed; however, their clinical or physiological significance cannot be determined within the scope of the present study. Similarly, the contribution of septal thickness parameters may reflect subtle geometric differences, although this requires confirmation in larger cohorts. In contrast, body weight, mid-septal strain, LVIDS, and basal posterior wall strain demonstrated minimal contribution to classification, indicating relative stability of these parameters within this dataset or potential redundancy once dominant longitudinal indices were incorporated. These findings do not establish independent associations but highlight patterns that may warrant further investigation in adequately powered studies. Parameters such as body weight and certain regional strain indices showed minimal contribution, although this may reflect limited statistical power rather than true absence of effect.

The machine learning component of this study was designed as an exploratory extension of conventional analysis. The XGBoost model achieved an AUC-ROC of 0.75 with high sensitivity; however, given the small sample size, lack of cross-validation, and absence of external validation, this performance should be interpreted as preliminary and not indicative of clinical applicability. Although the model showed improved discrimination compared with individual parameters, these differences should be viewed as descriptive rather than confirmatory. Similarly, calibration and decision curve analyses were performed on the same dataset used for model development and therefore do not provide independent evidence of model robustness. In human perioperative medicine, multivariable risk models are increasingly advocated for preoperative cardiac stratification [7,10,30]. Veterinary anesthesia guidelines likewise emphasize careful evaluation in geriatric patients, although evidence-based integration of advanced echocardiographic metrics remains limited [11,12]. The present findings suggest that combining deformation imaging with predictive modeling may enhance individualized perioperative management in dogs with mammary tumors. The findings should therefore be considered hypothesis-generating, providing preliminary insights that require validation in larger, independent cohorts before clinical implementation.

From a clinical perspective, the identification of subclinical myocardial dysfunction may be relevant in the context of preoperative evaluation. Mammary tumor surgery is categorized as non-cardiac; however, perioperative hemodynamic stress, anesthetic-induced myocardial depression, and inflammatory activation may unmask latent dysfunction [31,32]. The observed alteration in longitudinal function, not captured by conventional indices, highlights a potential cardiovascular vulnerability. However, as no perioperative or longitudinal clinical outcomes were evaluated, these findings should be interpreted as physiological observations rather than predictors of perioperative complications [33,34].

Several limitations must be acknowledged. First, this study was conducted at a single center and employed a retrospective design, which may limit generalizability and introduce inherent biases. Second, the relatively small sample size represents a major constraint, particularly in the context of predictive modeling. Limited sample size reduces statistical power, increases the risk of model instability and overfitting, and may lead to overly optimistic estimates of model performance. Therefore, the predictive results derived from the XGBoost algorithm should be interpreted with considerable caution and regarded as preliminary and hypothesis-generating rather than definitive. Although the XGBoost algorithm incorporates regularization techniques, these do not fully mitigate the risks associated with small datasets. The absence of internal cross-validation and external validation further limits the robustness and generalizability of the model. Third, age differences between groups represent a longitudinal parameter. The strict inclusion criteria used for the control group, requiring completely normal clinical, echocardiographic, and deformation parameters, may have introduced selection bias and could have exaggerated the differences between groups. Fourth, although frame rate variability was considered, the relatively wide range may still have influenced GLS measurements and should be acknowledged as a methodological limitation. Fifth, inflammatory (serum C-reactive protein) and cardiac biomarkers (e.g., cardiac troponin, NT-ProBNP, and CK) as well as tumor staging were not systematically evaluated, limiting mechanistic interpretation. Future studies should include larger, prospectively enrolled cohorts with appropriate validation strategies (including cross-validation and independent external validation), age-matched controls, standardized imaging protocols, and integrated biomarker profiling. Importantly, outcome-based validation is required to determine whether deformation-derived parameters or combined predictive models have true clinical and prognostic utility.

## 5. Conclusions

Dogs with mammary tumors exhibited alterations in longitudinal left ventricular function parameters, including MAPSE and GLS, despite preserved conventional systolic indices. These findings should be interpreted as preliminary and descriptive observations within the studied cohort. The integration of deformation imaging into a machine learning framework was technically feasible; however, the resulting model performance cannot be considered reliable in the absence of appropriate validation. Given the limited sample size, potential confounding factors, and lack of outcome data, the present results do not support definitive conclusions regarding myocardial dysfunction or perioperative cardiac risk. Accordingly, these findings should be viewed as exploratory and hypothesis-generating, providing a basis for future, adequately powered and validated studies. While similarities between canine mammary tumors and human breast cancer have been reported [3], any potential comparative or translational relevance remains speculative and requires confirmation in larger, independent cohorts.

## Figures and Tables

**Table 1:** Selected clinical and echocardiographic variables from healthy dogs and dogs with mammary tumors.

Parameter	Control (Mean ± SD)	Tumor (Mean ± SD)	*p* Value
**Physical examination**			
Age (yrs)	6.43 ± 2.30	10.00 ± 3.00	<0.05
Weight (kg)	18.50 ± 15.02	13.43 ± 7.19	NS
HR (bpm)	125.14 ± 15.13	133.22 ± 29.77	NS
**Conventional echocardiography**			
IVSD (mm)	8.86 ± 4.71	7.99 ± 1.54	NS
LVIDD (mm)	30.20 ± 11.18	27.47 ± 7.72	NS
LVPWD (mm)	8.36 ± 3.09	8.92 ± 2.15	NS
IVS (mm)	10.78 ± 4.58	10.56 ± 2.20	NS
LVIDS (mm)	20.46 ± 8.75	17.95 ± 6.50	NS
LVPWS (mm)	11.15 ± 3.49	12.77 ± 2.89	NS
EF %	62.00 ± 13.30	66.11 ± 12.23	NS
FS %	32.71 ± 9.21	35.78 ± 9.40	NS
LA (mm)	17.14 ± 9.62	19.44 ± 4.88	NS
AO (mm)	14.00 ± 7.75	17.89 ± 4.23	NS
LA/AO	1.05 ± 0.47	1.10 ± 0.12	NS
LA VOL (ml)	9.57 ± 8.22	6.44 ± 3.91	NS
LVIDDN (cm/kg)	1.37 ± 0.14	1.30 ± 0.19	NS
AO VMAX (m/s)	1.19 ± 0.47	1.02 ± 0.19	NS
MPA VMAX (m/s)	0.94 ± 0.24	0.89 ± 0.14	NS
MV E/A	1.15 ± 0.26	1.06 ± 0.30	NS
TV E/A	0.98 ± 0.46	0.96 ± 0.20	NS
TDI S’_sep_ (m/s)	0.11 ± 0.06	0.09 ± 0.02	NS
TDI S’_lat_ (m/s)	0.15 ± 0.10	0.09 ± 0.03	NS
MAPSE (cm)	1.04 ± 0.24	0.61 ± 0.26	<0.01
**LV tissue tracking and global and segmental strain analysis**			
TT_sep_ (mm)	4.39 ± 2.82	4.06 ± 1.32	NS
TT_lat_ (mm)	4.56 ± 3.34	4.54 ± 1.88	NS
GLS 4 CH %	−17.67 ± 1.55	−13.40 ± 3.76	<0.01
IVS Base %	−14.14 ± 6.49	−10.78 ± 2.44	NS
IVS Mid %	−18.00 ± 6.08	−16.44 ± 6.35	NS
IVS Apex %	−23.14 ± 8.51	−21.67 ± 7.57	NS
PW Apex %	−32.86 ± 7.71	−19.00 ± 8.34	<0.01
PW Mid %	−14.71 ± 5.94	−11.33 ± 4.33	NS
PW Base %	−10.71 ± 2.21	−11.33 ± 3.24	NS
GCS %	−14.6 ± 5.5	−17.6 ± 3.2	NS
GCSr (s^−1^)	−2.8 ± 0.8	−3.1 ± 1.1	NS

yrs, years; kg, kilogram; HR, heart rate (beat per minute); mm, millimeter, ml: milliliter; IVSD, interventricular septal thickness in diastole; LVIDD, left ventricular internal diameter in diastole; LVPWD, left ventricular posterior wall thickness in diastole; IVS, interventricular septum thickness; LVIDS, left ventricular internal diameter in systole; LVPWS, left ventricular posterior wall thickness in systole; EF, ejection fraction; FS, fractional shortening; LA, left atrial diameter; AO, aortic diameter; LA/AO, left atrium to aortic root ratio; LA VOL, left atrial volume; LVIDDN, normalized left ventricular internal diameter in diastole; AO VMAX, maximum aortic flow velocity; MPA VMAX, maximum main pulmonary artery flow velocity; MV E/A, mitral valve E/A ratio; TV E/A, tricuspid valve E/A ratio; TDI S’sep, tissue doppler systolic velocity of septal mitral annulus; TDI S’lat, tissue doppler systolic velocity of lateral mitral annulus; MAPSE, mitral annular plane systolic excursion; TTsep, tissue tracking displacement of septal LV wall; TTlat, tissue tracking displacement of lateral LV wall; GLS 4CH, global longitudinal strain from apical 4-chamber view; IVS Base, longitudinal strain of septum basal level; IVS Mid, longitudinal strain of septum mid-level; IVS Apex, longitudinal strain of septum apical level; PW Apex, longitudinal strain of posterior wall apical level; PW Mid, longitudinal strain of posterior wall mid-level; PW Base, longitudinal strain of posterior wall basal level; GCS, global circumferential strain; GCSr, global circumferential strain rate (s^−1^); NS, not significant.

## Data Availability

Original data are available from the first author (D.A.) upon request.

## Abbreviations

The following abbreviations are used in this manuscript:

2D-STE	Two-Dimensional Speckle-Tracking Echocardiography
Ao	Aorta
AO VMAX	Maximum Aortic Flow Velocity
AUC-ROC	Area Under the Receiver Operating Characteristic Curve
AutoEF	Automatic Ejection Fraction
CMTs	Canine Mammary Tumors
DCA	Decision Curve Analysis
ECG	Electrocardiography
EF	Ejection Fraction
FS	Fractional Shortening
GCS	Global Circumferential Strain
GCSr	Global Circumferential Strain Rate
GLS	Global Longitudinal Strain
HR	Heart Rate
IVS	Interventricular Septum
IVSd	Interventricular Septal Thickness in Diastole
IVSs	Interventricular Septal Thickness in Systole
IQR	Interquartile Range
KDE	Kernel Density Estimation
LA	Left Atrium
LA VOL	Left Atrial Volume
LA/Ao	Left Atrium to Aortic Ratio
LV	Left Ventricle
LVIDd	Left Ventricular Internal Diameter in Diastole
LVIDDN	Normalized Left Ventricular Internal Diameter in Diastole
LVIDs	Left Ventricular Internal Diameter in Systole
LVPWd	Left Ventricular Posterior Wall Thickness in Diastole
LVPWs	Left Ventricular Posterior Wall Thickness in Systole
MAPSE	Mitral Annular Plane Systolic Excursion
ML	Machine Learning
MPA VMAX	Maximum Main Pulmonary Artery Flow Velocity
MV E/A	Mitral Valve E/A Ratio
NS	Not Significant
PW	Posterior Wall
PW Doppler	Pulsed-Wave Doppler
R^2^	Coefficient of Determination
ROC	Receiver Operating Characteristic
SD	Standard Deviation
SHAP	SHapley Additive exPlanations
TDI	Tissue Doppler Imaging
TDI S’lat	Lateral Mitral Annulus Systolic Velocity
TDI S’sep	Septal Mitral Annulus Systolic Velocity
TT	Tissue Tracking
TTlat	Tissue Tracking Lateral Displacement
TTsep	Tissue Tracking Septal Displacement
TV E/A	Tricuspid Valve E/A Ratio
TVI	Tissue Velocity Imaging
XGBoost	eXtreme Gradient Boosting

## References

[1] Zatloukal J., Lorenzová J., Tichý F., Nečas A., Kecová H., Kohout P. (2005). Breed and Age as Risk Factors for Canine Mammary Tumours. Acta Vet. Brno.

[2] Dolka I., Czopowicz M., Stopka D., Wojtkowska A., Kaszak I., Sapierzyński R. (2024). Risk Factor Analysis and Clinicopathological Characteristics of Female Dogs with Mammary Tumours from a Single-Center Retrospective Study in Poland. Sci. Rep..

[3] Pastor N., Caballé N.C., Santella M., Ezquerra L.J., Tarazona R., Duran E. (2018). Epidemiological Study of Canine Mammary Tumors: Age, Breed, Size and Malignancy. Austral J. Vet. Sci..

[4] Vazquez E., Lipovka Y., Cervantes-Arias A., Garibay-Escobar A., Haby M.M., Queiroga F.L., Velazquez C. (2023). Canine Mammary Cancer: State of the Art and Future Perspectives. Animals.

[5] Andrew Novosad C. (2003). Principles of Treatment for Mammary Gland Tumors. Clin. Tech. Small Anim. Pract..

[6] Hörnfeldt M.B., Mortensen J.K. (2023). Surgical Dose and the Clinical Outcome in the Treatment of Mammary Gland Tumours in Female Dogs: A Literature Review. Acta Vet. Scand..

[7] Lee L.K.K., Tsai P.N.W., Ip K.Y., Irwin M.G. (2019). Pre-operative Cardiac Optimisation: A Directed Review. Anaesthesia.

[8] Mogensen J.E., Bach M.B.T., Bay P.G., Varlik T., Willesen J.L., Gleerup C.H., Koch J. (2025). Speckle-Tracking Echocardiography in Dogs: Evaluating Imaging Parameters and Methodological Variability in Global Longitudinal Strain Assessment. Animals.

[9] Yilmaz Z., Salci H., Levent P., Algan D., Varlik T., Topçu M.E., Tanaka R., Hamabe L. (2025). Thromboelastographic Assessment of Coagulation Profiles in Dogs with Cardiac Tumors and Their Relationship to Cardiac Function. Animals.

[10] Abraham S.A., Eagle K.A. (1994). Preoperative Cardiac Risk Assessment for Noncardiac Surgery. J. Nucl. Cardiol..

[11] Bustamante R., González-Pérez E., Caro-Vadillo A., Aguado D. (2024). Impact of Preanaesthetic Electrocardiogram on Decision Making and Modification of Anaesthetic Protocols in Dogs. Vet. Rec..

[12] Grubb T., Sager J., Gaynor J.S., Montgomery E., Parker J.A., Shafford H., Tearney C. (2020). 2020 AAHA Anesthesia and Monitoring Guidelines for Dogs and Cats*. J. Am. Anim. Hosp. Assoc..

[13] Marques M.G., Marques A.E.G.W., de Siqueira C.E., de Sousa É.A.P., Ribeiro Y.S., Floriano B.P., Ferreira W.L., Santos P.S.P. (2020). Conventional Echocardiography and Two-Dimensional Speckle Tracking in Healthy Sevoflurane-Anesthetized Dogs Undergoing Continuous Rate Infusion of Nalbuphine. Vet. Med. Int..

[14] Hamabe L., Mandour A.S., Shimada K., Uemura A., Yilmaz Z., Nagaoka K., Tanaka R. (2021). Role of Two-Dimensional Speckle-Tracking Echocardiography in Early Detection of Left Ventricular Dysfunction in Dogs. Animals.

[15] Huang S.J., Ting I., Huang A.M., Slama M., McLean A.S. (2017). Longitudinal Wall Fractional Shortening: An M-Mode Index Based on Mitral Annular Plane Systolic Excursion (MAPSE) That Correlates and Predicts Left Ventricular Longitudinal Strain (LVLS) in Intensive Care Patients. Crit. Care.

[16] Dickson D., Shave R., Rishniw M., Patteson M. (2017). Echocardiographic Assessments of Longitudinal Left Ventricular Function in Healthy English Springer Spaniels. J. Vet. Cardiol..

[17] Algan D., Varlik T., Koch J., Yilmaz Z. (2025). Diagnostic and Prognostic Significance of Platelet Large Cell Count and Ratio in Cats across Stages of Hypertrophic Cardiomyopathy. Res. Vet. Sci..

[18] De Madron E., Bussadori C., Chetboul V., De Madron E. (2016). Clinical Echocardiography in the Dogs and Cats.

[19] Thomas W.P., Gaber C.E., Jacobs G.J., Kaplan P.M., Lombard C.W., Vet M., Moise N.S., Moses B.L. (1993). Recommendations for Standards in Transthoracic Two-Dimensional Echocardiography in the Dog and Cat. J. Vet. Intern. Med..

[20] Varlik T., Algan D., Sönmez Ö., Singh K.K., Ülger Ö., Kubat G.B., Koch J., Yilmaz Z. (2026). Intravenous Mitochondrial Transplantation as an Adjunctive Therapy for Dilated Cardiomyopathy. Mitochondrion.

[21] Cerbu M., Cerbu C., Papuc I. (2023). M-Mode Echocardiography in Canine Veterinary Practice: A Comprehensive Review of Left Ventricular Measurements in 44 Different Dog Breeds. Animals.

[22] Cornell C.C., Kittleson M.D., Torre P.D., Häggström J., Lombard C.W., Pedersen H.D., Vollmar A., Wey A. (2004). Allometric Scaling of M-Mode Cardiac Measurements in Normal Adult Dogs. J. Vet. Intern. Med..

[23] Santarelli G., Baron Toaldo M., Bouvard J., Glaus T.M., Fernández del Palacio J. (2019). Variability among Strain Variables Derived from Two-Dimensional Speckle Tracking Echocardiography in Dogs by Use of Various Software. Am. J. Vet. Res..

[24] Li M., Fu X., Li D. (2020). Diabetes Prediction Based on XGBoost Algorithm. IOP Conf. Ser. Mater. Sci. Eng..

[25] Fernández-Parra R., Tissier R., Alvarado M.P., Garde-Sanjuan L., Verwaerde P., Saponaro V. (2021). Conventional and Advanced Echocardiographic Assessment of Systolic Function in Dogs Sedated with Dexmedetomidine or Acepromazine. Res. Vet. Sci..

[26] Kouris N.T., Kostopoulos V.S., Psarrou G.A., Kostakou P.M., Tzavara C., Olympios C.D. (2021). Left Ventricular Ejection Fraction and Global Longitudinal Strain Variability between Methodology and Experience. Echocardiography.

[27] Nakanishi K., Daimon M. (2022). Aging and Myocardial Strain. J. Med. Ultrason..

[28] Schick A.L., Kaine J.C., Al-Sadhan N.A., Lin T., Baird J., Bahit K., Dwyer K.H. (2023). Focused Cardiac Ultrasound with Mitral Annular Plane Systolic Excursion (MAPSE) Detection of Left Ventricular Dysfunction. Am. J. Emerg. Med..

[29] Corda A., Pinna Parpaglia M.L., Sotgiu G., Zobba R., Gomez Ochoa P., Prieto Ramos J., French A. (2019). Use of 2-Dimensional Speckle-Tracking Echocardiography to Assess Left Ventricular Systolic Function in Dogs with Systemic Inflammatory Response Syndrome. J. Vet. Intern. Med..

[30] Huang L., Lin B., Mu Y., Ren Y., Li Q., Li Y., Ma Y., Fan Y., Zhu G., Song Z. (2025). Associations of Inflammation-Related Hematological Profile with the Early-Stages of Cardiovascular-Kidney-Metabolic Syndrome and the Mediating Role of Body Composition: Evidence from the China National Health Survey. Diabetol. Metab. Syndr..

[31] van Trigt P., Christian C.C., Fagraeus L., Spray T.L., Peyton R.B., Pellom G.L., Wechsler A.S. (1984). Myocardial Depression by Anesthetic Agents (Halothane, Enflurane and Nitrous Oxide): Quantitation Based on End-Systolic Pressure-Dimension Relations. Am. J. Cardiol..

[32] Djuric M., Nenadic I., Radisavljevic N., Todorovic D., Stojanovic M., Dimic N., Bobos M., Bojic S., Stevanovic P., Savic P. (2025). The Influence of Anesthetics on the Functions of the Endothelium and Oxidative Stress: A Critical Review. Biomedicines.

[33] Glas K.E., Swaminathan M., Reeves S.T., Shanewise J.S., Rubenson D., Smith P.K., Mathew J.P., Shernan S.K. (2007). Guidelines for the Performance of a Comprehensive Intraoperative Epiaortic Ultrasonographic Examination: Recommendations of the American Society of Echocardiography and the Society of Cardiovascular Anesthesiologists; Endorsed by the Society of Thoracic Surgeons. J. Am. Soc. Echocardiogr..

[34] Raslau D., Bierle D.M., Stephenson C.R., Mikhail M.A., Kebede E.B., Mauck K.F. (2020). Preoperative Cardiac Risk Assessment. Mayo Clin. Proc..

